# Balance between Regulatory T and Th17 Cells in Systemic Lupus Erythematosus: The Old and the New

**DOI:** 10.1155/2012/823085

**Published:** 2012-06-14

**Authors:** Alessia Alunno, Elena Bartoloni, Onelia Bistoni, Giuseppe Nocentini, Simona Ronchetti, Sara Caterbi, Valentina Valentini, Carlo Riccardi, Roberto Gerli

**Affiliations:** ^1^Rheumatology Unit, Department of Clinical and Experimental Medicine, University of Perugia, Via Enrico dal Pozzo, 06122 Perugia, Italy; ^2^Section of Pharmacology, Toxicology and Chemotherapy, University of Perugia, Via Enrico dal Pozzo, 06122 Perugia, Italy

## Abstract

Pathogenic mechanisms underlying the development of systemic lupus erythematosus (SLE) are very complex and not yet entirely clarified. However, the pivotal role of T lymphocytes in the induction and perpetuation of aberrant immune response is well established. Among T cells, IL-17 producing T helper (Th17) cells and regulatory T (Treg) cells represent an intriguing issue to be addressed in SLE pathogenesis, since an imbalance between the two subsets has been observed in the course of the disease. Treg cells appear to be impaired and therefore unable to counteract autoreactive T lymphocytes. Conversely, Th17 cells accumulate in target organs contributing to local IL-17 production and eventually tissue damage. In this setting, targeting Treg/Th17 balance for therapeutic purposes may represent an intriguing and useful tool for SLE treatment in the next future. In this paper, the current knowledge about Treg and Th17 cells interplay in SLE will be discussed.

## 1. Introduction

Systemic lupus erythematosus (SLE) is an autoimmune disorder affecting almost all organs and tissues [1, 2]. In genetically predisposed subjects, environmental factors, such as viral infections and smoking, induce the breakdown of self-tolerance eventually triggering autoimmune response [1, 2]. The clinical heterogeneity of the disease often represents a challenge for clinicians and reflects the complexity of underlying pathogenic mechanisms. The aberrant crosstalk between different immune cells such as B and T lymphocytes represents a milestone in the natural history of SLE and, in general terms, of all autoimmune conditions. Self-antigen presentation by antigen presenting cells may be identified as the *primum movens *that leads to recruitment, activation, and expansion of autoreactive lymphocytes. This cascade culminates with disease-specific autoantibody production by B cells and eventually with target tissue injury [1–3]. B lymphocytes are well-recognized actors in SLE pathogenesis, and this is further confirmed by the effectiveness of B-cell depleting therapies in these patients [4]. Moreover, an altered T-cell homeostasis which plays a pivotal role in the development of the disease and the longstanding paradigm of T helper (Th) 1/Th2 cell immune response was recently challenged by the recognition of Th17 cells and regulatory T cells (Treg) [5, 6]. This intriguing evidence pointed out the need to call into question previous discoveries. The aim of this paper is to discuss the current knowledge about the interplay between Treg and Th17 cells in the pathogenesis of SLE and potential therapeutic intervention in this setting.

## 2. Th17 Cell Subsets in SLE

Th17 cells were identified according to their capability to produce IL-17, and initially they were thought to be just a variant of Th1 cells and to origin from a common precursor [5, 7]. Nonetheless, further investigation ruled out this possibility and found that Th17 commitment of naïve T cells, by the expression of STAT-3 and retinoic acid orphan receptor-(ROR-) *γ*t, was attributable to the presence of both transforming growth factor-(TGF) *β* and IL-6 in the surrounding microenvironment [8–12].

First cloned in 1995, IL-17 family cytokines display a broad spectrum of action including the capacity to induce the production of inflammatory and tissue-damaging molecules.

In particular, IL-17A is able to stimulate the production of chemokines and cytokines from multiple cells like epithelial cells and fibroblasts [13] and to promote the proliferation, maturation, and recruitment of neutrophils, macrophages, and lymphocytes through the induction of colony-stimulating factors and chemokines [13]. 

Concerning IL-17 in SLE, recent data from humans and mice clearly support the role of this cytokine and Th17 cells in lupus pathogenesis. To note, it has been observed by several groups that SLE patients, including those with new-onset disease, had increased serum or plasma levels of IL-17, expansion of IL-17-producing T cells in the peripheral blood, and infiltration of Th17 cells in target organs like the kidneys [14–20]. Some studies showed that circulating IL-17 levels correlate with disease activity [14–17] and are associated with kidney involvement [20]. In addition, increased expression of IL-17 and ROR*γ*t mRNA has been found in urine sediments from lupus patients [21]. Taken together, these results point out the pivotal role played by IL-17 in mediating target organ damage in both early and long-standing stages of the disease [22].

Notably, the involvement of IL-17 in the perpetuation of lupus nephritis was recently underlined by the elegant study performed by Crispín et al., in which a novel pathogenic Th17 cell subset was identified [23]. This small T-cell population, named double negative (DN) according to the lack of both CD4 and CD8 molecules expression, appeared to be responsible for most of IL-17 production in sera and kidney of SLE patients. This may be explained by the hypothesis that all DN T cells are already committed *in vivo* towards a Th17 phenotype, whereas CD4^+^ cells require additional stimuli to differentiate into IL-17 producing T cells. Interesting, IL-17 can also promote humoral immunity that plays a major role in lupus pathogenesis. IL-17, alone or in combination with B-cell activating factor (BAFF), increases the survival and proliferation of human B cells as well as the differentiation of B cells into antibody-producing cells [12, 24]. In conclusion, IL-17 could promote inflammation in lupus by affecting both cellular and humoral immune response. In this setting, additional studies are needed to find out the mechanisms for increased Th17 cell response and the therapeutic implication of targeting IL-17 in SLE, as discussed in detail below.

## 3. Treg-Cell Subsets in SLE

The other face of the coin in the pathogenesis of SLE is represented by Treg cells [25]. Since their first identification in late 90s, Treg cells became a hot topic in immunology because of the recognition of a link between impairment of this cell population and development of autoimmunity [6, 26–29]. Indeed, Treg cells display suppressive activity towards autoreactive lymphocytes thus preventing the onset of aberrant self-immune response [30]. Initially, Treg-cells were isolated in humans and mice according to high surface levels of CD25 (IL-2R*α*) and intracellular expression of the forkhead winged helix (Fox) P3 transcription factor. FoxP3 expression is required for commitment of Treg cells and maintenance of their functional activity [31]. To date, several Treg cell subsets have been identified. They differ from each other for either phenotypic features or origin. Natural Treg cells (nTreg) are produced in the thymus in the very early phases of life following an appropriate T-cell receptor (TCR) stimulation and in the presence of a peculiar cytokine microenvironment. Conversely, inducible Treg cells (iTreg) result from the differentiation of naïve T cells in secondary lymphoid organs during the entire life [32, 33]. However, this classification appears slightly restrictive nowadays, since deeper understanding of Treg cell physiology has been achieved. Our group and other investigators observed that T cells lacking CD25 and expressing FoxP3 displayed suppressive activity towards effector T cells from both healthy subjects and pathological conditions [34–37] Interestingly, we provided the clue that the surface expression of glucocorticoid-induced tumor necrosis factor receptor family-related protein (GITR) on CD25^−^ T cells was able to confer them a regulatory phenotype and function [34]. In addition, as will be discussed in detail below, iTreg cells may originate from activated T cells when appropriate stimuli are present in the surrounding microenvironment [38]. Abnormalities of this fine tuning may result in the development of autoimmunity.

In recent years, Treg-cell assessment in SLE has been performed by several groups [39]. Unfortunately, although much effort has been spent to shed some light on Treg imbalance in SLE, conclusive data are still lacking [40]. The majority of these studies reported either reduced number or impaired function of circulating Treg cells in SLE [41–44]. On the opposite, others failed to observe any abnormalities in this T-cell subset and found resistance of effector T cells to regulatory activity of Treg cells [45, 46]. These discrepancies may arise from fair differences between isolation protocols and flow-cytometry technicalities resulting in strong difficulties in comparing results from different studies. Moreover, data regarding Treg cells in SLE target organs, such as kidney, are poor, and, hence, regulatory mechanisms controlling Treg homeostasis within affected tissues are still a matter of debate [47, 48]. According to the aforementioned data regarding putative Treg cells lacking CD25, Zhang et al. recently evaluated circulating CD25^−^ T cells in SLE patients and surprisingly found an increase of the CD25^−^FoxP3^+^ fraction [49]. Further analysis, however, allowed to conclude that this cell subpopulation was actually divergent from conventional Treg cells, as they failed to exert suppressive activity *in vitro* towards CD25^−^FoxP3^−^ effector T cells [50]. In this setting, albeit FoxP3 is universally accepted as specific marker of Treg cells, it must be taken into account that in some cases its expression may be misinterpreted. Noteworthy, it has been recently suggested that, besides the expression itself, FoxP3 intensity is the true discriminator between effector and regulatory T cells. Indeed, FoxP3^low^ T cells often produced IL-2 independently on CD25 surface levels, whereas FoxP3^high^ T cells did not [51]. Furthermore, we demonstrated that among the CD25^−^ cell subset, only those coexpressing GITR on the surface display *in vitro *suppressive activity that can be reverted by antibody-mediated GITR blockade [34]. In conclusion, the definite role of Treg-cells in SLE is still uncertain and further studies are required to shed some light on this controversial issue. At the same time, the identification of more specific phenotypic Treg cell markers in an attempt to minimize variability between different studies are surely needed.

## 4. The Interplay between Th17 and Treg Cells in SLE: Who's Who?

Besides the above-mentioned difficulty to clarify the effective role played by Th17/Treg cells in SLE pathogenesis, recent studies made this matter even more complex providing the clue of a plasticity between the two T-cell subsets [38]. As suggested by Lee et al., it appeared that TGF-*β* plays a dual role on naïve T cells depending on the presence or absence of IL-6. The combination of TGF-*β* and IL-6 allows the differentiation toward a Th17 phenotype, whereas if TGF-*β* is present alone, iTreg cells will be generated [10, 11, 52]. Furthermore, transition between Th17 and Th1 cells may also be possible. Indeed, several groups have generated Th17 cells *in vitro* and adoptively transferred to induce autoimmune disease in mice. After transfer *in vivo*, Th17 cells quickly acquire the ability to produce IFN-*γ*, as Th1 cells do, and lose their capacity to release IL-17 [53, 54]. A similar behavior was observed *in vitro* following several culture rounds. It has been proposed that the shift from Th17 to Th1 cell may be due, at least in part, to the fact that Th17 cells express IL-12 receptors and readily produce IFN*γ* in response to IL-12 exposure [55]. In addition, also an epigenetic mechanism may underlie the plasticity of CD4^+^ T helper cell differentiation, as recently suggested [56].

These findings draw our attention to new intriguing scenarios in which the cytokine milieu is the key player that globally drives immune response towards either health or disease [57]. However, it has been already described that SLE flares may occur as a consequence of cytokine imbalance and eventually of the Th17/Treg ratio in lupus-prone mice [8]. More recently, it has been demonstrated that such imbalance is not limited to SLE flares but is hallmark of the disease, since also patients with quiescent disease display a Th17/Treg ratio favoring Th17 cells [58, 59]. Taken together, these evidences prompt therapeutic approaches aimed to restore an adequate cytokine network and Th17/Treg balance in SLE. Indeed, although Th17 cells play a key role in the pathogenesis of the disease, Th1 and other effector cells are also involved in the perpetuation of autoimmune response [14, 60–63]. Therefore, selective Th17 targeting may not be sufficient to counteract chronic inflammation in SLE patients. On the other hand, restoring the immune balance between Th17 and Treg cells may help to achieve a better clinical response. These observations are strengthened by the evidence that selective Th17 blockade led to disease worsening in a murine model of colitis and exacerbated acute graft-versus-host disease, reasonably for a rebound increase of Th1 cells [64, 65]. In this context, it is noteworthy that previous studies have confirmed the ability of some agents, such as 2,3,7,8-tetrachlorodibenzo-*p*-dioxin (TCDD), rapamycin, and the vitamin A metabolite all-trans-retinoic acid, to promote the conversion of Th17 to Treg cells in experimental autoimmune encephalomyelitis [66–68]. It is to note, however, that TCDD is toxic in animals and humans, while all-trans-retinoic acid and rapamycin have not been yet tested in humans. Moreover, the nucleosomal histone peptide epitope H471-94 appears to be able to induce generation of Treg cells and suppression of inflammatory Th17 cells in lupus-prone mice, through the induction of tolerogenic dendritic cells rather than via a direct effect on Treg/Th17 cells [69].

In conclusion, the aforementioned data suggest that targeting Th17 and Treg cells for therapeutic purposes in SLE may be possible. However, further investigations aimed to identify well-tolerated and powerful compounds that induce the diversion of T-cell differentiation from Th17 cells to Treg cells are needed [70, 71].

## 5. Conclusions

Aberrant T-cell homeostasis is a crucial event in SLE pathogenesis, and Th17/Treg imbalance appears to represent an important key pathogenic player. However, many aspects of such a deregulation in the course of the disease are still uncertain, and conclusive data about specific underlying mechanisms are lacking. Promising results concerning therapeutic targeting Th17/Treg cell balance may open new lines of investigation for SLE treatment in the near future.
